# Commentary: Organ Cultures for Retinal Diseases

**DOI:** 10.3389/fnins.2021.714094

**Published:** 2021-07-23

**Authors:** Stephen R. Mut, Maribel Vazquez

**Affiliations:** Department of Biomedical Engineering, Rutgers, The State University of New Jersey, New Brunswick, NJ, United States

**Keywords:** microfluidics, vision loss, *in vitro*, stem cells, regeneration

The review by Hurst et al. (2020) is a comprehensive article published within this journal about the use of organotypic culture systems as models to study retinal diseases. The article noted that use of microfluidic technologies, such as microelectrode arrays (MEAs), can be significant in measuring cellular activity within organ culture systems (Hurst et al., 2020). An additional emerging area for microfluidics is their integration with explants to enrich transplantation strategies used to treat retinal degenerative diseases.

Progressive vision loss in adults is escalating worldwide, as the incidence of macular degeneration and diabetic retinopathy are expected to exceed 300 million and 642 million, respectively, by 2040 (Mitchell et al., 2018; Simo-Servat et al., 2019). The retina consists of a varied network of neurons that synapse with one another across three nuclear layers. Damage to any one type of neuron within this intricate network propagates dysfunction to result in progressive vision loss.

Contemporary cell replacement therapies offer exciting promise to restore vision by replacing damaged neurons with transplanted stem cells. Numerous platforms have been developed to elucidate the cellular mechanisms able to promote stem cell integration within mature retinal hosts (Wu et al., 2018). However, ongoing projects have produced mixed results, including low stem cell survival and the inability of stem cells to differentiate and/or position themselves appropriately within the retinal network (Gokoffski et al., 2019). A variety of *in vitro* and organotypic platforms have been developed to examine native stem cell behaviors within microscale environments (reviewed in Greene et al., 2019). Surprisingly, few of these projects have incorporated microfluidic technologies to model cues from damaged adult retina, such as fields of injury cytokines and degraded cellular matrixes (reviewed in Vazquez, 2020), that differ significantly from stem cell environments. A current thrust is to bridge microfluidic technologies with explanted retina to develop hybrid, quantitative models to examine stem cell behaviors within adult, organotypic cultures.

Initial hybrid models (Figure 1) cultured retinal explants within micro-scale transwell systems to measure long-term viability (Rettinger and Wang, 2018), while newer models integrated microfluidic perfusion systems for controlled delivery of neurotransmitters and therapeutics (Dodson et al., 2015; Rountree et al., 2017). Most recently, our group developed a hybrid system called the Ex Vivo Eye Facsimile System (EVES) to examine how extrinsic factors, such as chemical and electrical gradients, can promote appropriate stem cell positioning within retinal hosts (Mishra et al., 2019; Vazquez et al., 2020). Our system consists of a 3D environment that can be rapidly prototyped to meet the geometric constraints of enucleated eyes derived from a variety of animal models. Our preliminary EVES studies illustrated that combined electrochemical fields increased the numbers of motile stem cells and the distances migrated within rodent eye facsimiles. The integration of microfluidics with organotypic retinal cultures will therefore produce a new generation of quantitative platforms that enable newfound applications of external fields to enrich stem cell replacement strategies.

**Figure 1:**
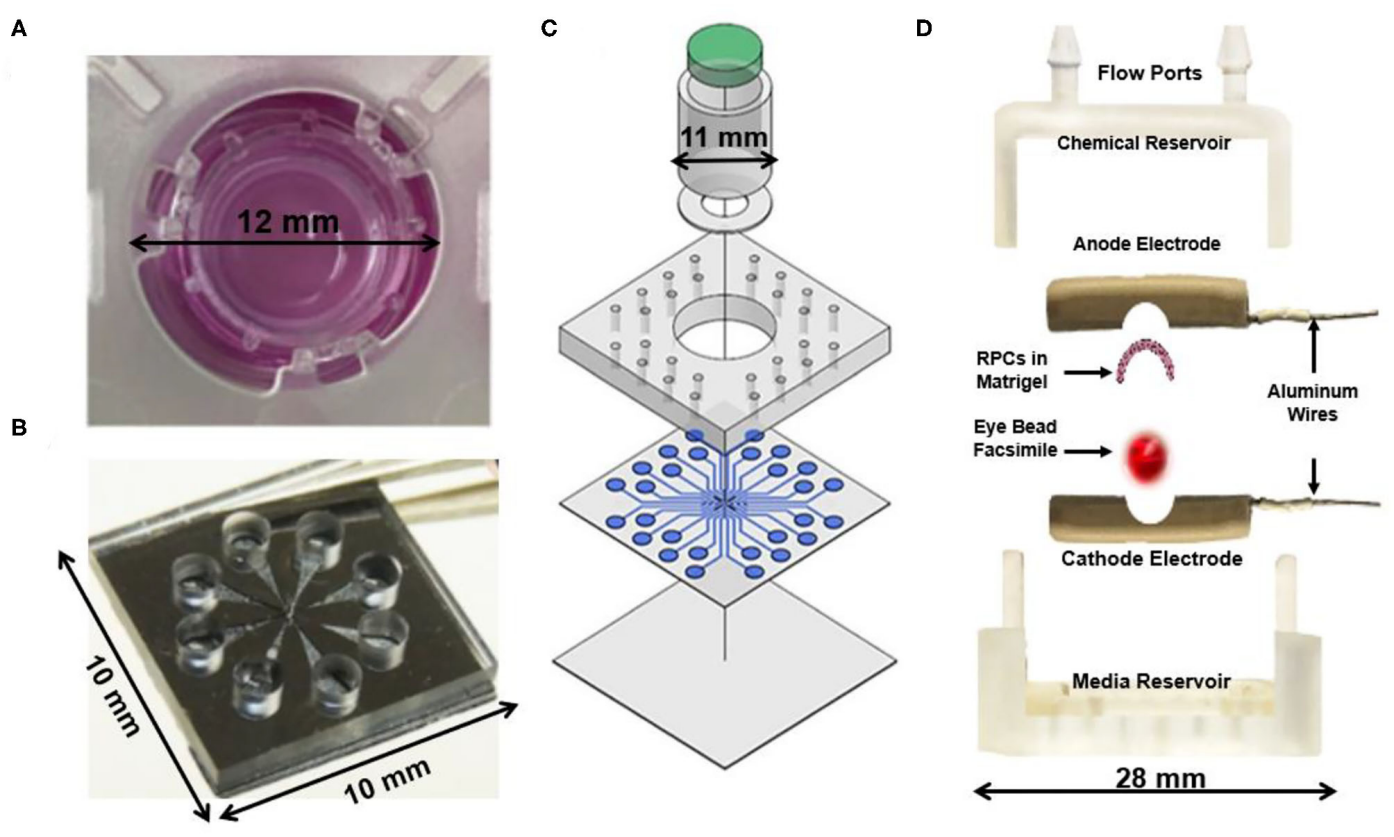
Current hybrid explant systems for studying the retina. **(A)** An *ex vivo* chamber for a porcine retina explant capable of maintaining tissue viability for 1–2 weeks to enable long-term investigation of the *ex vivo* retina (Rettinger and Wang, 2018). **(B)** A glutamate perfusion system to stimulate retinal neurons with neurotransmitters to augment the phototransduction process that occurs within the body (Rountree et al., 2017). **(C)** Retina on a chip microfluidic perfusion assay system for point access delivery of therapeutics to specific parts of the rat retina (Dodson et al., 2015). **(D)** *Ex Vivo* Eye Facsimile (EVES) hybrid explant system designed for delivery of electrical and chemical stimulation to a whole-enucleated eye explant from mouse (Vazquez et al., 2020).

## Author Contributions

SM developed and wrote the manuscript. MV wrote and provided edits to the manuscript. All authors approved the final manuscript version for publication.

## Conflict of Interest

The authors declare that the research was conducted in the absence of any commercial or financial relationships that could be construed as a potential conflict of interest.

## Publisher's Note

All claims expressed in this article are solely those of the authors and do not necessarily represent those of their affiliated organizations, or those of the publisher, the editors and the reviewers. Any product that may be evaluated in this article, or claim that may be made by its manufacturer, is not guaranteed or endorsed by the publisher.
